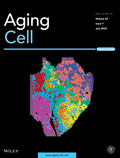# Featured Cover

**DOI:** 10.1111/acel.13931

**Published:** 2023-07-17

**Authors:** Ombretta Garbarino, Luca Lambroia, Gianluca Basso, Veronica Marrella, Barbara Franceschini, Cristiana Soldani, Fabio Pasqualini, Desiree Giuliano, Guido Costa, Clelia Peano, Davide Barbarossa, Destro Annarita, Andreina Salvati, Luigi Terracciano, Guido Torzilli, Matteo Donadon, Francesca Faggioli

## Abstract

Cover legend: The cover image is based on the Research Article *Spatial resolution of cellular senescence dynamics in human colorectal liver metastasis* by Ombretta Garbarino et al., https://doi.org/10.1111/acel.13853